# Factors influencing nutrition education in elementary schools: A qualitative study

**DOI:** 10.1177/00178969251386236

**Published:** 2025-12-03

**Authors:** Beatriz Franco-Arellano, Ellen Vogel, Matthew Stein, Jacqueline Marie Brown, Vanessa Laketa, JoAnne Arcand

**Affiliations:** a Faculty of Health Sciences, Ontario Tech University, Oshawa, ON, Canada; b Centennial College, Toronto, ON, Canada

**Keywords:** Canada, elementary schools, health interventions, implementation research, nutrition education, teachers

## Abstract

**Background:**

Curriculum-based nutrition education is a common strategy to build food literacy skills and promote lifelong healthy eating. While past research has reported on the barriers teachers face when teaching about nutrition, very few recent studies have been published on the topic. This paper aimed to explore some of the views, barriers and facilitators that teachers face when teaching nutrition in elementary schools.

**Methods:**

A descriptive qualitative research study was conducted among elementary school teachers in Ontario, Canada, using a semi-structured interview guide. The guide was informed by the Consolidated Framework for Implementation Research, and then validated and pilot tested. Two independent researchers inductively coded and thematically analysed the transcripts.

**Results:**

Participants (*n* = 9) indicated that nutrition was an important part of their teaching. Barriers to teaching nutrition included insufficient training/professional development opportunities, limited instructional time, competition with other subjects deemed a higher priority, limited financial resources to pay for teaching materials, lack of French language resources and sensitivities related to culture, socioeconomic status, and eating disorders. In contrast, interactive learning activities, integrating nutrition with other subjects, and support from school leadership, parents and professional organisations were seen as facilitators of nutrition education.

**Conclusions:**

Enhancing teacher training, resources and support while prioritising nutrition in the school curriculum is crucial for effective and equitable nutrition education in Canadian elementary schools. Addressing barriers and leveraging the facilitators identified in this study are essential to improve curriculum-based nutrition education provision.

## Introduction

Comprehensive school nutrition programmes have been shown to enhance food literacy, eating habits and diet quality (Ashoori et al., 2023; Aydin et al., 2021; Chaudhary et al., 2020; Park et al., 2022). A fundamental component of school nutrition programmes is curriculum-based nutrition education, which has a goal of developing food literacy skills. Nutrition education via the curriculum positively impacts children’s nutrition knowledge, dietary habits and health (Canadian Pediatric Society, 2020; Metos et al., 2019). Such interventions are considered beneficial by parents and teachers alike, and have become increasingly important as opportunities for developing food literacy skills in the home have declined due to reduced family meal times and less time spent preparing food (Aydin et al., 2021; Slater, 2017).

Despite its benefits, however, teachers often experience challenges teaching nutrition to students. Research internationally has found that time and other curriculum requirements are among the predominant barriers for teachers implementing nutrition education (Aydin et al., 2021; Hastert et al., 2022; Olarte et al., 2022). In contrast, the availability of guidelines and lesson plans, age-appropriate activities and strong parental engagement can support nutrition education (Murimi et al., 2018). Although past research has shed some light on factors impacting nutrition education in classrooms, there are no contemporary studies that have comprehensively assessed the different factors influencing nutrition education implementation. These include curriculum factors, teacher-specific factors (e.g. knowledge, motivation), school factors (e.g. school support, resources, available training) and other external influences (Hudson et al., 2020; Leeman et al., 2018; McLoughlin et al., 2020; Wendt et al., 2023).

This study addresses these research gaps by exploring views, barriers and facilitators that influence nutrition curriculum implementation among Canadian elementary school teachers.

## Methods

### Participants

A descriptive qualitative research study of teachers was conducted via online focus groups (90 minutes) and individual interviews (30 minutes) between August 2022 and March 2023. This design was chosen to gain a deep understanding of teachers’ lived experiences with nutrition education, including an understanding of barriers and facilitators faced when doing so. Focus groups were the primary method of data collection to provide participants with the opportunity to share their experiences and encourage richer discussion; individual interviews were used in cases where we were unable to schedule multiple participants. Participants were certified elementary school teachers (Kindergarten to Grade 8) working in Ontario, Canada. Study participants were purposively sampled by expressing interest in the study through social media advertisements, which was chosen as the method of recruitment to reach eligible teachers from across the province. The research team, the STEAM Lab at Ontario Tech University and the Ontario Physical Health Education Association (OPHEA) advertised the study on their Facebook social media platforms, with a link to an online sign-up form. This study was reviewed by the Ontario Tech University research ethics board (Reference: 16608), and a written informed consent was obtained prior to data collection. All participants who agreed to complete the study were included in the analysis. The researchers had no prior relationships with the interviewees. A sample size of 12 participants was anticipated to reach theoretical saturation, and participants were recruited until saturation was achieved.

### Instrumentation

A semi-structured interview guide with probes was developed using the Consolidated Framework for Implementation Research (CFIR) and relevant literature (Cho and Nadow, 2004; Damschroder et al., 2009, 2022; Domínguez Rodríguez et al., 2022; Lee and Gortmaker, 2012; Lukas and Cunningham-Sabo, 2011; Patte and Leatherdale, 2016; Porter et al., 2018; Wachira and Keengwe, 2011; Walker, 2019).

The CFIR is an implementation science framework that has been used in similar studies and allows for the exploration of implementation factors across five domains. In this case, it allowed for an examination of the factors impacting the implementation of nutrition education (Hudson et al., 2020; Leeman et al., 2018; McLoughlin et al., 2022; Meshkovska et al., 2022; Sobers et al., 2021; Wendt et al., 2023). These include the *innovation domain* (e.g. quality of the content and advantages of nutrition education), the *inner setting domain* (e.g. the setting in which the innovation is implemented such as access to resources and school leadership), the *outer setting domain* (e.g. the setting where the inner setting exists like school curricula and competing interests), the *individuals domain* (e.g. the roles and characteristics of the individuals involved in the innovation including elements of confidence and preparedness), and the *implementation process domain* (e.g. the activities and strategies used to implement the innovation, namely, relative significance and challenges).

**Figure 1.**
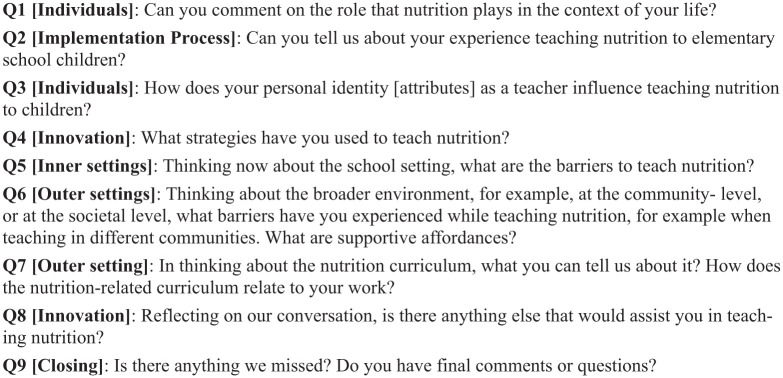
Semi-structured interview guide.

The final interview guide contained nine questions with probes that were reviewed and face and content validated by nutritionists and teachers (Figure 1). The guide was pilot tested with one elementary school teacher who had no previous relationship with the researchers. After pilot testing, the guide was modified to improve question clarity and include some of the linguistic terms most used by teachers. For example, the original interview guide referred to teaching ‘approaches’ and this term was changed to ‘strategies’ based on pilot testing feedback.

### Procedure

All interviews were held online using Zoom and audio recorded. Prior to the study, participants were sent the meeting link and an instruction sheet containing practical information to prepare prior the call. On the day of the focus groups or individual interviews, the facilitator (BFA) greeted participants, provided the instructions for the session, started the recording and led the discussion. A notetaker (JMB) took detailed notes during the meeting, including their observations, non-verbal cues and summaries of participant responses. At the end of each session, the facilitator summarised key themes present in the interview with the participant(s), who had the opportunity to provide any additional insights. The facilitator and notetaker debriefed after each session and the lead author (BFA) generated a session summary. The notes from the notetaker were used to complement the anonymised summary of each focus group or individual interview that was then sent to participants individually for member checking. No comments or revisions were received from participants during member checking. Participants were offered a $50 CAD gift card honorarium as compensation for their time and any expenses incurred. Throughout the recruitment and data collection process, trustworthiness was sought by building rapport with participants through clear communication, ensuring transparency of the research and consent process, and using group moderation strategies in focus groups to ensure all voices were captured.

### Data analysis

Audio recordings were transcribed verbatim (BFA, VL) and de-identified using code numbers. Coding was conducted by two researchers (BFA, MS) who independently coded each of the verbatim files to generate themes. One author (BFA) merged the coded files with the initial themes, which were reviewed by both authors. If discrepancies emerged, they were discussed among both (BFA, MS) researchers and agreed upon over two rounds. A codebook was generated for both authors (BFA, MS) to review and refine salient themes. Although the CFIR guided the development of the interview guide, the themes were inductively generated. The detailed notes taken during the interviews were triangulated against the transcripts during the data analysis process. NVivo (v.14, QSR International) was used to organise data.

## Results

Nine participants were interviewed in two focus groups (*n* = 6, with 3 participants per focus group, sessions averaged 90 minutes) or individual interviews (*n* = 3, interviews averaged 30 minutes). A tenth participant who agreed to the study dropped out before starting one of the sessions due to illness. Although saturation (i.e. ‘conceptual depth’) was reached after two focus groups and two interviews, a third individual interview was conducted, which confirmed the findings.

Participants (women, 100%) had diverse backgrounds in terms of number of years of teaching experience (range = 10–30 years), the grades taught (Kindergarten-12), the type of school worked in (public school, which is funded by the government; or independent school, which is paid for by parents or guardians), language of instruction (English, French) and educational background (Education, Health Studies). Two participants were Physical Education teachers who were responsible for teaching the nutrition unit in multiple classrooms. Most (*n* = 8) participants taught in public schools, but two taught or had taught in independent elementary schools. At the time the study was conducted, all the participants were teaching in the Province of Ontario; however, some participants had previously taught in other regions of Canada.

Participants expressed that healthy eating was an instrumental part of their daily personal lives. There was a strong agreement that nutrition was a very important subject for children: ‘Nutrition is really important you have to feed your body to feed your brain’ (Teacher 4). Overall, all participants (*n* = 9) were enthusiastic and passionate about teaching nutrition and found that children positively respond to health and nutrition teaching and learning units.

### Prioritisation and time available to teach nutrition

There was a strong agreement among all nine participants about the lack of emphasis in the school curriculum on nutrition education, compared to other subjects. Participants felt the nutrition curriculum was too separate and too briefly mentioned at all grades, ‘Our nutrition curriculum is five classes, five classes in the entire year!’ (Teacher 8), and was particularly limited at the lower grades (i.e. Kindergarten-Grade 2). All participants strongly felt that nutrition, health and physical education were often not prioritised: ‘Oftentimes, health is kind of lower on the priority list and sometimes it gets pushed aside’ (Teacher 6). This view was echoed by another teacher:

I think the problem really stems from lack of emphasis in our curriculum. . .There is such a small component of the health curriculum, and then the health curriculum is a part of the whole physical education curriculum, so it’s like a small part of a small part. (Teacher 1)

In addition, lack of time was seen as a barrier to the introduction of nutrition concepts taught in the classroom, largely due to competing interests with other subjects such as mathematics and language:

So, when you have such limited time, you’re not going to be able to cover anything beyond the curriculum. . . we also don’t have any mandatory hours. . .the only thing that we have for mandatory hours is math and French. (Teacher 1)

### Resources and infrastructure to teach nutrition

The majority of participants (*n* = 6) described the limited availability of nutrition teaching resources. They recognised that the Ontario provincial website provided very few teaching resources and no ‘quick and easy to use’ materials. Participants noted that most of the resources available to them were non-interactive and sometimes not friendly for classroom use. While participants recognised that curriculum documents were not meant to be lesson plans, there was a need for more guidance on how to implement the nutrition curriculum: ‘I don’t know who makes these [curricula] up, but they have not been in the classroom for very. . . for a long time’ (Teacher 4).

Ideas and resources on how to implement curricular content for each grade were welcomed:

Like that’s something that the government did by example for the maths, they included links that you can go and you can see examples, so that something maybe we can add a little bit. (Teacher 3)

Another teacher concurred,

I kind of wish that there was like a little more in terms of guidance with the curriculum, perhaps. Like, you know they tell you what to do but like not really how to do it necessarily. (Teacher 6)

In French-speaking schools, the most important barrier was access to suitable French language resources. One participant reported using nutrition-related resources obtained from other provinces (e.g. from Quebec). However, these resources usually required adaptation for grade-level appropriateness: ‘there’s not that much in French, . . . [and] it has to be adapted to the level that the kids can understand. . .’ (Teacher 3).

Most teachers sought out classroom materials independently (*n* = 6). Professional networks (e.g. social media, teachers’ groups) were important sources participants went to to find classroom materials. Some teachers used online platforms to find teaching resources, but the resources on them were not always free. Procuring them posed an additional barrier to access. As one participant suggested, ‘I’m always having to buy something to support my curriculum’ (Teacher 4). Another participant discussed the financial burden for teachers: ‘I was just reflecting on the same thing, how that financial burden . . . falls on the classroom teacher’ (Teacher 7). Five participants found high costs and lack of funding as a barrier to the ability to organise field trips (e.g. to farms) that would provide an engaging avenue for nutrition education.

One participant considered the lack of dedicated classroom space a significant barrier to teaching about nutrition. For example, teaching nutrition in a shared gymnasium while other groups are having a physical education class was a suboptimal learning environment. As one participant stated,

. . .we’re doing health in the gym, as well and you know, if it’s a split gym and you hear the noise from another [class] and it’s in a gym and you’re kind of like . . . well I don’t really want to bring food and prep food in here. (Teacher 7)

Two participants commented that while schools may have technological devices (e.g. laptops, tablets) for students to use during class, the resources were rarely available for physical education teachers. For example, one participant stated that devices needed to be booked well in advance: ‘they are very rarely available, so you really have to know in advance when are you booking them’ (Teacher 9).

### Nutrition-specific education and training

Several participants felt that having a physical education or science background would be beneficial when teaching nutrition (*n* = 5). They also noted that school board mandated training specific to nutrition was largely non-existent: ‘I feel yeah we really need some PD [professional development] . . . [teachers] never really had the specific kind of classes or training that I feel they need to. . .’ (Teacher 9). Physical education teachers commonly acquired nutrition knowledge informally from peers by sharing knowledge or materials. For instance, one participant described how health and nutrition training materials for teachers had been developed at one school, but then the programme had been suspended due to funding cuts:

A couple of years ago, like for five years, I was the educational consultant for my school board, so I was training teachers, and one of the subjects I was in charge [of] was the gym teachers and the health part of it, so it was nice because I work with a teacher that was still in class and we actually developed like a little activity programme for the teachers’ resource. That was nice but that [programme] was cut, so I was there for 5 years, but after that they decided that gym and health that was not important, I guess. . . (Teacher 3)

There was agreement among participants (*n* = 6) that having professional development specific to nutrition for teachers would be helpful, and that this training should be provided within school hours. One participant suggested that pairing experienced teachers with novice teachers could encourage training of educators and facilitate knowledge transfer.

### Social factors

Students’ cultural background and the community in which schools are situated were important factors influencing teaching about nutrition. One participant described how some children struggled to identify the difference between fruit and vegetables: ‘I guess they did not grow up with certain aspects of nutrition and they don’t really know what’s healthy, what’s unhealthy, or what’s the “sometimes” food’ (Teacher 9). Participants (*n* = 8) agreed that nutrition could be a sensitive topic for a number of students, and it was important to use language that does not reflect negatively on students or their families:

You also have to respect the families and the different you know background, and sometimes they don’t have the possibility to have fruits and things like that, so it’s delicate I find. (Teacher 3)

Talking about the same issue, another participant indicated that it was critical to be aware of cultural differences:

The reality is I’m [of] a certain culture and so I think about when I’m talking about nutrition and different types of foods, is it culturally relevant? Is it culturally applicable? (Teacher 7)

All nine participants were mindful about food affordability, food insecurity and difficulty in access to healthy food options when teaching nutrition. They also reported stark differences between schools with different socioeconomic statuses, with one stating,

And what I noticed was that communities in that are more affluent, when I talk about food and nutrition, the students would say we talk about in terms of like when you’re shopping for food, buy organic. . . In areas where the community has a lower literacy rate and economic status. . . I’ve practically like never heard them mention anything about organic food. (Teacher 1)

One teacher found that including food insecurity as a topic in nutrition classes was a positive strategy to teach how food access and affordability could be a barrier to healthy eating, especially to students from affluent families that might have never experienced food insecurity. Two participants were also concerned about discussing nutrition and food choices when there are students who have or are at higher risk of eating disorders.

### Interactive learning

Participants agreed that real-life activities such as field trips to grocery stores were the best strategies to use when teaching nutrition, regardless of the grade. Hands-on activities were seen as engaging and meaningful to students. For example, one participant described a learning activity in which students created a mock restaurant, developed a menu and parents were later invited to ‘order’ food at the restaurant. This activity was integrated into other subjects such as language (writing the menu) and mathematics (adding up the bill and working out the change to be given to parents).

Participants indicated that children responded very well to technology-based classes, and were more engaged when mobile devices were integrated into the classroom. While some participants (*n* = 4) preferred to use non-technology-based activities for nutrition education, they recognised that there are very few credible technology-based resources for nutrition education. The quality of the content was the most important factor to take into account when deciding to use a nutrition resource, whether technology or non-technology based: ‘It doesn’t matter whether it is technology, if the content is good, it’s interesting, there is a capture for my kids, then I’ll use it, I will learn it’ (Teacher 1).

Other strategies that facilitated learning about nutrition include food logs, food preparation competitions, and field trips. Guest lectures led by dietitians were suggested as another engaging approach to interest children: ‘It’s always interesting to have a nutritionist person coming in the class and assist us in a few lessons’ (Teacher 3), and ‘The kids are really engaged when we have guest speakers’ (Teacher 9).

### Integrating nutrition with other subjects

Most participants (*n* = 7) reported having experience integrating nutrition into other subjects including financial literacy, language (e.g. food blogs), mathematics (e.g. teaching fractions with fruit), environment, geography and physical education (e.g. running in the gym towards different fruit or vegetable ‘groups’):

I think it’s great to integrate into other subjects. . . I found that the kids were more engaged just simply because I was integrating it with math, with language, like other subjects like geography as well. (Teacher 1)

Working collaboratively with colleagues was considered important especially when the classroom and physical education teachers had shared responsibility for the nutrition unit: ‘The past year we had a new gym teacher and [this teacher] was excellent and did lot of things, and it was nice we worked together as a team’ (Teacher 3). To close gaps, participants suggested the nutrition curriculum could be delivered throughout the school year and shared with other teachers too. Two participants also indicated that students would benefit if home economics classes were reintroduced into schools. These classes are not currently available in Ontario.

### Supportive factors internal and external to the school environment

School leadership by principals and other senior staff was considered to be both a barrier and a facilitator for teaching nutrition. Some participants (*n* = 4) had found school leadership to be a very supportive experience. One of them said, ‘Our principal is very open . . . she is very accommodating’ (Teacher 9); another concurred, ‘The principal is very open minded and very receptive, like if we come with a project, she’ll find the money and help us’ (Teacher 3).

Other important facilitators were third-party organisations such as Health Canada; the Ontario Physical and Health Education Association (OPHEA), a registered provincial charity supporting the implementation of health and wellness initiatives in Ontario schools; and Teachers Pay Teachers, an online marketplace where teachers buy and sell original educational materials. Almost all participants (*n* = 8) reported using the lesson plans and video/audio resources provided by these organisations. Participants mentioned the value of having high-quality resources that aligned with curricula and that came from credible sources. One of them said, ‘I love the way the Canada Food Guide (CFG) is now in a plate. . . I found it really easy for them to just actually visualise what that should look like’ (Teacher 6). Another said,

I always went to OPHEA because it had very clear ideas, very clear lesson plans, very easy to execute. They didn’t require a lot of time on my hands to prepare or think about or, like, they would have a worksheet or just an activity, and it was all really well done, like it was really user-friendly. (Teacher 5)

Finally, many participants (*n* = 6) reported finding strong support from their students’ families, regardless of the type of community they worked in. Connecting nutrition to students’ home life was felt to be extremely important:

I find they [the parents] actually appreciated the meal prep at home assignment that we’ve done. Parents seem to love that. . . So, I find they’re generally supportive of [nutrition education]. (Teacher 8)

## Discussion

This study sought to explore the delivery of nutrition education in elementary school classrooms in Ontario, Canada. The use of the CFIR allowed the research team to delve deeply into topics related to teachers themselves, the schools in which they work and external factors that might enable or hinder teaching and learning practices in relation to nutrition. In the course of the study, we found that teachers faced several challenges when teaching nutrition.

Our findings align with those in the wider literature revealing how a lack of teaching resources may impact teachers’ confidence to teach nutrition (Aydin et al., 2021). These barriers are exacerbated by the lack of funding to support the development and delivery of school nutrition programmes. Importantly, since this study was conducted, the Canadian government has announced the launch of a national school food programme, effectively ending the status of Canada as the only G7 country without a federal school nutrition programme (Government of Canada, 2024). Health Canada also published a toolkit for educators, which can provide guidance when teaching nutrition to young people, although classroom resources still remain limited (Health Canada, 2024).

Despite the barriers, our study found that teachers were highly enthusiastic about teaching nutrition. This finding broadly supports research from the USA that found elementary school teachers have positive attitudes towards nutrition and recognise its important impact on health (Metos et al., 2019). Teachers in our study suggested that interactive activities were among the best to provide nutrition education. This supports literature indicating children learn better when playing, and fun, hands-on learning activities can enhance nutrition education in schools (Kelly and Nash, 2021). Participants in our study also suggested that health professionals such as nutritionists or Registered Dietitians can assist in the delivery of nutrition education, a finding supported by the wider literature (Metos et al., 2019).

Furthermore, participants suggested that it is critical to involve parents as they can also support nutrition classes (e.g. as guests in schools), as suggested by Aydin et al. (2021). Teachers consistently reported the need to engage parents in school health efforts (Hastert et al., 2022), but also recognised the challenges and sensitivities associated with discussing food and nutrition with parents, such as differing opinions about rewarding children with sweets (Aydin et al., 2022). To ease these challenges, teachers have used newsletters and other teacher–parent channels to communicate information about food and nutrition programmes, as well as nutrition classes (Aydin et al., 2022). With respect to cultural integration, our findings align with a recent scoping review which suggested that effective food literacy interventions require the integration of the cultural and emotional aspects of food choice and their relevance to the local community (Kelly and Nash, 2021).

### Implications for school health policy, practice and equity

Currently, theory-based school nutrition programmes are not commonly used in Ontario schools. Likewise, there is a need to provide nutrition-specific training and professional development to teachers and schools (Olarte et al., 2022). Collaborations with government and policymakers are crucial to update curricula and to create implementation support, such as teaching tools and resources, to enhance the provision of nutrition education. While initiatives like Canada’s national school food programme and the Food Guide toolkit are promising first steps, further research into the effectiveness of classroom-based nutrition education is essential. Universities should consider integrating nutrition training into initial teacher education and training programmes, and partnerships with experts, such as Registered Dietitians can lead to the co-creation of teaching materials and training for educators. Future research is needed to evaluate teacher nutrition training to ensure it is effective in improving both teacher-related outcomes (e.g. self-efficacy and confidence in teaching nutrition) and student learning.

### Limitations and strengths

There are a number of limitations to this study. First, while the sample size was small, it was consistent with that identified as appropriate in the literature (Hennink and Kaiser, 2022) and allowed us to reach theoretical saturation, as determined by no new information, themes or insights arising from the data. In order to reach out to teachers in the aftermath of COVID-19, the study used a combination of focus groups and individual interviews. This approach was a necessity since recruitment and scheduling proved a challenge at a time when the workload demands placed on teachers had increased. The study was conducted online instead of in-person, which may have reduced the richness of data elicited from participants (Carter et al., 2021). However, online interviews have also been shown to offer benefits that we observed in this study, such as reducing geographical barriers, accommodating participants’ schedules and observing whether participants followed the researcher’s instruction to allow everyone to finish speaking and respect other people’s time (Earnesty et al., 2022; Fielding et al., 2017; Moore et al., 2015).

## Conclusion

This study identified several barriers to implementing nutrition curriculum in schools in Ontario, including insufficient training opportunities; limited nutrition-related classroom teaching materials; teacher concerns regarding appropriate discussion of sensitive food-related issues; and limited teaching time dedicated to nutrition due to competition with other high-priority subjects. In contrast, interactive learning activities, integrating nutrition education with other subjects and support from school leadership, parents and professional organisations were seen as enabling nutrition education. These insights underscore the need for targeted strategies and interventions to overcome barriers, enhance food literacy and improve children’s and young people’s health outcomes.
